# Higher mineralized bone volume is associated with a lower plain X-Ray vascular calcification score in hemodialysis patients

**DOI:** 10.1371/journal.pone.0179868

**Published:** 2017-07-07

**Authors:** Teresa Adragao, Anibal Ferreira, Joao M. Frazao, Ana Luisa Papoila, Iola Pinto, Marie-Claude Monier-Faugere, Hartmut H. Malluche

**Affiliations:** 1 Nephrology Department, Santa Cruz Hospital, Lisbon, Portugal; 2 Nephrology Department, Curry Cabral Hospital, Lisbon, Portugal; 3 Nephrology Department, S Joao Hospital, Medical School and Nephrology Research and Development Unit, University of Porto, Porto, Portugal; 4 CEAUL, CEDOC, Nova Medical School/FCM, New University, Lisbon, Portugal; 5 ISEL, CMA FCT-UNL, Lisbon, Portugal; 6 Division of Nephrology, Bone and Mineral Metabolism, University of Kentucky, Lexington, KY, United States of America; Universidade Estadual Paulista Julio de Mesquita Filho, BRAZIL

## Abstract

**Background and objectives:**

In dialysis patients, there is an increasing evidence that altered bone metabolism is associated with cardiovascular calcifications. The main objective of this study was to analyse, in hemodialysis patients, the relationships between bone turnover, mineralization and volume, evaluated in bone biopsies, with a plain X-ray vascular calcification score.

**Design, setting, participants and measurements:**

In a cross-sectional study, bone biopsies and evaluation of vascular calcifications were performed in fifty hemodialysis patients. Cancellous bone volume, mineralized bone volume, osteoid volume, activation frequency, bone formation rate/bone surface, osteoid thickness and mineralization lag time were determined by histomorphometry. Vascular calcifications were assessed by the simple vascular calcification score (SVCS) in plain X-Ray of pelvis and hands and, for comparison, by the Agatston score in Multi-Slice Computed Tomography (MSCT).

**Results:**

SVCS≥3 was present in 20 patients (40%). Low and high bone turnover were present in 54% and 38% of patients, respectively. Low bone volume was present in 20% of patients. In multivariable analysis, higher age (p = 0.015) and longer hemodialysis duration (p = 0.017) were associated with SVCS≥3. Contrary to cancellous bone volume, the addition to this model of mineralized bone volume (OR = 0.863; 95%CI: 0.766, 0.971; p = 0.015), improved the performance of the model. For each increase of 1% in mineralized bone volume there was a 13.7% decrease in the odds of having SVCS≥3 (p = 0.015). An Agatston score>400 was observed in 80% of the patients with a SVCS≥3 versus 4% of patients with a SVCS<3, (p<0.001).

**Conclusion:**

Higher mineralized bone volume was associated with a lower plain X-ray vascular calcification. This study corroborates the hypothesis of the existence of a link between bone and vessel and reinforces the clinical utility of this simple and inexpensive vascular calcification score in dialysis patients.

## Introduction

Vascular calcifications are associated with cardiovascular morbidity and mortality in dialysis patients [1–4]. It has been demonstrated that vascular calcification in dialysis and non-dialysis patients is an active cellular process, similar to bone formation [5–7]. In an experimental model, high levels of phosphorus and calcium [5] are some of the factors that activate the core binding factor α-1 (Cbfa-1), which is the first step for the differentiation of vascular smooth muscle cells into osteoblasts [5]. Reduction of calcification inhibitors, such as Fetuin-A, matrix-Gla protein [8] and pyrophosphate [9,10] may be another factor associated with the development of calcification. In dialysis patients, there is increasing evidence that altered bone metabolism is associated with cardiovascular calcifications.

KDIGO has recommended in 2006 a new classification for mineral and bone disorders of chronic kidney disease that includes the presence of vascular calcifications [11]. Low bone turnover [12–14] and low bone volume [15] have been associated with vascular calcifications. Adynamic bone disease [13] and osteoporosis have been associated with progression of coronary calcifications [16]. An association between osteoporotic fractures, vascular calcifications and mortality has been demonstrated in dialysis patients [17]. We have developed a simple vascular calcification score (SVCS) evaluated in plain X-Ray of pelvis and hands, which is a predictor of cardiovascular mortality and cardiovascular disease in dialysis patients [3,18,19] and in CKD patients not on dialysis [20]. The main objective of this study was to evaluate, in dialysis patients, relationships between histomorphometric parameters of bone turnover, mineralization and volume with this simple vascular calcification score. The secondary objective was to evaluate, in the same patients, the agreement between the SVCS and the Agatston score obtained by multislice computed tomography (MSCT).

## Material and methods

### Study design

**Fig 1:**
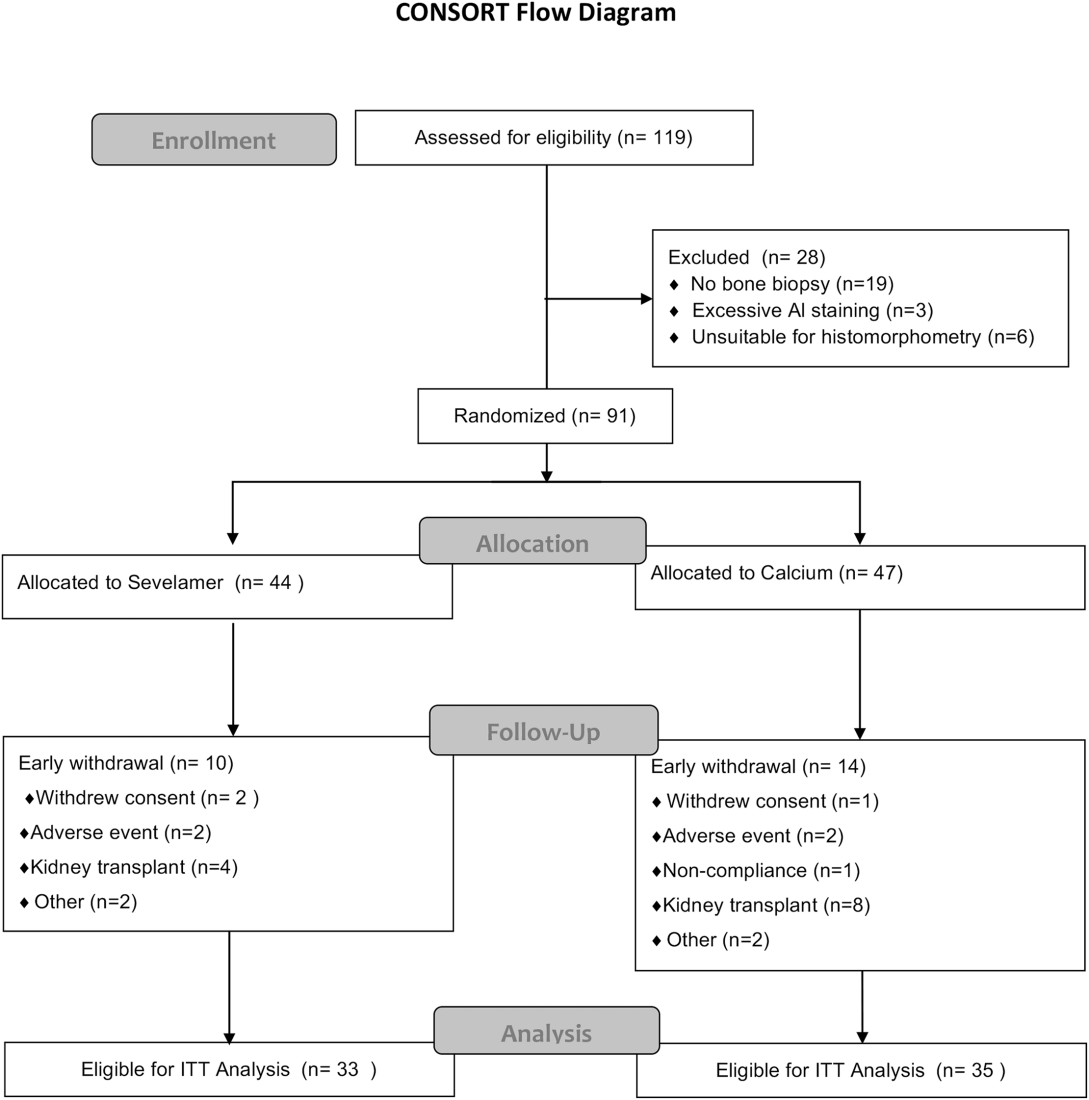
The intention-to-treat (ITT) population was defined as all patients who were randomly assigned, received one or more doses of study medication, and had a second bone biopsy. One patient in the sevelamer group completed treatment but did not have a second bone biopsy and so was excluded from the ITT analysis. Two patients in the calcium group withdrew from the study early but received one or more doses of study medication and had a second bone biopsy and so were included in the ITT analysis.

This is a cross-sectional study performed in a group of prevalent hemodialysis patients to evaluate relationships between histomorphometric parameters of bone, evaluated by bone biopsy, with vascular calcifications evaluated by plain X-ray (SVCS). This is an extension study performed in a cohort of 50 patients participating in a European randomized clinical trial, with the Study Number: GTC-68-402, that analysed the effect of sevelamer hydrochloride and calcium carbonate on bone turnover and mineralization in hemodialysis patients [21], (Fig 1). This randomized clinical trial was a requisition of EMA (European Medicines Agency) and was initiated in 2002. This trial has no registration number because The European Clinical Trials Register started after 1 May 2004 with the creation of EdraCT.

Our present study was an extension study from this clinical trial and included a group of 50 patients that accepted undergo evaluation of vascular calcifications in the same diagnostic center, in Lisbon, immediately after the second bone biopsy performed in the clinical trial. The protocol of this study was approved by the institutional ethical committees of the hemodialysis units participating in the study (FMC Entroncamento, Torres Vedras and Barreiro; CMDR, Centro Médico de Doenças Renais, HPA, Hospital Particular Almada, SPD, Sociedade Portuguesa de Diálise, Uninefro, Santo Tirso and NefroNorte, Paredes). All patients signed an informed consent, and all the studies procedures were in accordance with the recommendations for research involving human beings of the Helsinqui Declaration (with the amendments of Tokyo 1975, Hong-Kong 1989, Somerset West 1996 and Edimburg 2000) and of the WHO).

### Assessment of vascular calcifications

Vascular calcifications were assessed by the simple vascular calcification score (SVCS), a semi-quantitative score developed by us and evaluated in plain radiographs of pelvis and hands [3]. Pelvis films were divided into four sections by two imaginary lines: a horizontal line over the upper limit of both femoral heads and a median vertical line over the vertebral column. A horizontal line divided hand films over the upper limit of the metacarpal bones. In each section, the presence of any type of vascular calcification lining the vessel walls, either in a linear or irregular pattern, was rated as 1 and its absence as 0. Final score was the sum of calcifications found in all sections and ranged from 0 to 8. In the same diagnostic centre, vascular calcifications were simultaneously assessed in 42 patients by the Agatston score, using Multislice Computed Tomography (MSCT). MSCT scans were performed with the four-slice technique on the model Somatom Volume Zoom (Siemens AG, Erlangen, Germany). Slices of 2.5 mm thickness were acquired under the following conditions: 120 kVp, 130 mAs, and 0.5 gantry rotation time. All images were transferred to a workstation and analysed with calcium scoring software (HeartView CT, Siemens AG, Erlangen, Germany).

Plain X-Ray calcifications and coronary Agatston score were evaluated at the same diagnostic center, 3.8 ±1.9 months after bone biopsy.

### Bone biopsies and bone histomorphometry

All bone biopsies were performed in Lisbon (by A.F.) and Oporto (by J.M.F). Anterior iliac crest bone biopsies were done after tetracycline double labelling under local anesthesia and conscious sedation. The labelling schedule consisted of a 2 days oral administration of tetracycline hydrochloride (250 mg twice daily) followed by a drug-free interval of 10 days, and subsequent oral administration of demeclocycline hydrochloride (300 mg twice daily) for 4 days. Bone biopsies were performed 3 to 4 days after completing the second label. Bone samples were obtained with the one-step electrical drill technique (Straumann Medical, Waldenburg, Switzerland). All bone samples were processed and analysed at the Bone Diagnostic and Research Laboratory, University of Kentucky, (Lexington, KY) without knowledge of the clinical data.

Iliac crest bone samples were fixed in ethanol, dehydrated, and embedded in methylmethacrylate as previously described [22]. Serial sections of 3 and 7-micrometer thickness were cut with a Microm microtome equipped with a carbide edged knife (HM360, Microm, Walldorf, Germany). Sections were stained with the modified Masson-Goldner trichrome stain [23] and for aluminium detection with the aurin tricarboxylic acid stain [24], and solochrome azurine stain [25]. Unstained sections were prepared for phase contrast and fluorescent light microscopy. Histomorphometry for parameters of bone structure and bone remodelling was done using the Osteoplan II system (C. Zeiss, NY) [26,27]. Bone turnover was determined by activation frequency (Ac.f) (Normal 0.49–0.72/year) and bone formation rate per bone surface (BFR/BS) (Normal 1.8–3.8 mm^3^/cm^2^/year). Bone mineralization was evaluated by mineralization lag time (Mlt) (Normal < 50 days) and osteoid thickness (O.Th) (Normal 4–20 μm). Bone volume was evaluated as cancellous bone volume per tissue volume (BV/TV) (Normal 16–23%), mineralized bone volume per tissue volume (Md.BV/TV) (Normal 13–21%) and osteoid volume per bone volume (OV/BV) (Normal 0.57–6.00%). Bone histomorphometry nomenclature and units are in agreement with the 2012 update report of the ASBMR histomorphometry nomenclature committee [28]. Normal values are from American and European studies. These values have been used in prior studies including histomorphometric assessment of bone from patients of Portugal and other European countries and from the USA [15,21,29–34].

### Biochemical analysis

Serum levels of the following biochemical parameters were evaluated and time averaged for the 12 months preceding the bone biopsy: serum calcium, phosphorus, and iPTH were evaluated every 4 weeks; bone specific alkaline phosphatase, 25-(OH)-vitamin D_3_; 1,25-(OH)_2_-vitamin D_3_ and lipid profile were evaluated every 6 months; iPTH was evaluated using the DPC Immulite PTH IRMA; the reference range is 25 to 87 pg/mL and the intra and inter-assay coefficients of variation are <7% and <9%, respectively; 1,25-(OH)_2_-vitaminD_3_ and 25-(OH)-vitamin D were analysed by radioimmunoassay using LIAISON kits (DiaSorin, Sallugia, Italy). The reference range for 1,25-(OH)_2_-vitaminD_3_ was 25 to 86.5 pg/mL and the intra and inter-assay coefficients of variation were <2.9% and <5.9%, respectively. The reference range for 25-(OH)-vitaminD_3_ was 25 to 100 ng/mL and the intra and inter-assay coefficients of variation were 4.1% and 7% respectively. Total cholesterol, LDL-cholesterol, HDL-cholesterol and triglycerides were measured every 4 months by the Synchron LX system (Beckman Coulter, Fullerton, California).

### Statistical analysis

An exploratory analysis was carried out for all variables. Categorical data were presented as frequencies and percentages, and continuous variables as mean ± standard deviation or median and inter-quartile range (25^th^ percentile; 75^th^ percentile). Univariable analysis was done using Student´s t-test and nonparametric tests (Chi-square, Fisher’s Exact, Mann-Whitney U) whenever outliers and skewed distributions were present.

The multivariable analysis was performed using logistic regression models where the dependent variable was SVCS after being dichotomized (SVCS<3 and SVCS≥3), according to previously demonstrated association with cardiovascular mortality (3,18). To obtain a first model (model 1), all the variables with a p-value<0.25 obtained in the univariable analysis and with clinical relevance, were considered. To compare bone parameters regarding their association with vascular calcifications measured by SVCS, three additional models were adjusted. These models were obtained by adding to model 1, separately, the variables BV/TV (bone volume, model 2), Md.BV/TV (mineralized bone volume, model 3) and OV/BV (osteoid volume, model 4). Bone volume consists of mineralized bone volume and osteoid volume.

To quantify the improvement resulting from adding BV/TV, (Model 2), Md.BV/TV (Model 3) and OV/BV (Model 4) to Model 1, continuous net reclassification improvement (NRI) and integrated discrimination improvement (IDI) measures were calculated [35,36] (supplemental statistical analysis, S1 Table). Predictiveness curves were also calculated [37].

To study the agreement between SVCS and Agatston score, Spearman’s correlation coefficient was estimated. A further agreement analysis, considering both SVCS and Agatston scores after being dichotomized, was performed and Cohen’s kappa was obtained with corresponding bias corrected accelerated bootstrap 95% confidence intervals. The interpretation of kappa is the following: values≤0 indicate no agreement, 0.01–0.20 slight, 0.21–0.40 fair, 0.41–0.60 moderate, 0.61–0.80 substantial, and 0.81–0.99, almost perfect agreement [38,39]. In this analysis, for Agatston score the cut-point 400 was considered [40]. For SVCS, the cut-point that best discriminated lower (≤400) from higher Agatston scores (>400) was determined using minimum p-value approach. Based on a systematic search for the best cut-point, the point that is associated with the minimum chi-squared test p-value or, equivalently, with the maximum chi-squared test value in a grid of marker values is identified [41]. Sensitivity, specificity, positive predictive value, and negative predicted value were calculated for the obtained cut-point.

Confidence intervals (95% CI) were also calculated, as required. The level of significance α = 0.05 was considered.

All data were analyzed using SPSS 22.0 (*IBM Corp*. *Released 2013*. *IBM SPSS Statistics for Windows*. *Armonk*, *NY*: *IBM Corp*) and R software (R: A Language and Environment for Statistical Computing, R Core Team, R Foundation for Statistical Computing, Vienna, Austria, 2014).

## Results

Fifty patients (27 men and 23 women) from the above-mentioned randomized trial [21] were enrolled, with a mean age of 53.52 ± 15.61 years and a median hemodialysis duration of 36.87 months (P_25_ = 25.95; P_75_ = 65.15). The only inclusion criterion for the present study was the patient’s agreement to undergo evaluation of vascular calcifications in a single centre which was performed at 3.8 ± 1.9 months after bone biopsies.

Phosphate binders were given during the year before bone biopsies either with sevelamer hydrochloride in 26 patients, 52%; mean dose 4.1 ± 1.9 g/day or calcium carbonate in 24 patients, 48%; mean dose 3.7 ± 1.8 g/day. Twenty-nine patients (58%) were treated with active vitamin D with a mean dose 3.8 ± 2.7 μg/week. Eight patients (16%) were smokers and five (10%) were diabetic.

The demographic, biochemical, bone summary measures of the study sample and their association with SVCS are presented in Tables 1 and 2, respectively. Vascular calcifications were detected by plain X-ray in 30 patients (60%). Pelvic vascular calcifications were present in 29 patients (58%) and hand vascular calcifications were present in 16 patients (32%). SVCS ≥3 was found in 20 patients (40%). Low and high bone turnover were present in 27 patients (54%) and 19 patients (38%); there were no cases of osteomalacia. None of the bone biopsies showed positive stain for aluminium.

**Table 1:** Univariable analysis: Demographic, biochemical and vascular calcification parameters.

Variables	All patients(n = 50)	SVCS<3 (n = 30; 60%)	SVCS≥3 (n = 20; 40%)	p value
Age (years)	53.52 ± 15.61	49.63 ± 15.63	59.35 ± 14.00	0.030
Male gender n(%)	27 (54%)	12 (40%)	15 (75%)	0.021^(a)^
Diabetes n(%)	5 (10%)	0 (0%)	5 (25%)	0.007^(b)^
Smoking habits n(%)	8 (16%)	5 (17%)	3 (15%)	1.000^(b)^
HD duration (months)	36.87 (25.95; 65.15)	34.12 (25.80; 51.76)	44.72 (26.79; 93.13)	0.089 ^(c)^
Hb (mg/dL)	12.57 ± 3.46	11.99 ± 1.00	12.96 ± 4.38	0.339
Ca (mg/dL)	9.56 ± 0.62	9.44 ± 0.51	9.73 ± 0.74	0.111
P (mg/dL)	5.04 ± 0.86	4.94 ± 0.94	5.19 ± 0.73	0.329
iPTH (pg/mL)	284 (186; 551)	310 (215; 590)	236 (160; 495)	0.259^(c)^
25 (OH) vit D_3_ (ng/mL)	19.6 (13.4; 24.8)	20.4 (14.0;24.8)	16.4 (12.4; 24.7)	0.382^(c)^
1.25(OH)_2_vit D_3_ (pg/mL)	11.91 ± 8.25	12.66 ± 9.90	10.78 ± 4.86	0.435
Total cholesterol (mmol/L)	1.64 ± 0.27	1.66 ± 0.28	1.62 ± 0.26	0.593
LDL cholesterol (mmol/L)	0.99 ± 0.26	1.00 ± 0.27	0.97 ± 0.24	0.658
HDL cholesterol (mmol/L)	0.48 ± 0.12	0.46 ± 0.11	0.51 ± 0.12	0.100
Triglycerides (mmol/L)	1.76 (1.30;2.37)	1.58 (1.25;2.25)	2.02 (1.55;3.10)	0.154^(c)^
Calcium Carbonate n(%)	24 (48%)	16 (54%)	8 (40%)	0.399^(a)^
Agatston score>400 (n = 42)	n = 42	n = 27	n = 15	<0.001^(b)^
	13 (31%)	1 (4%)	12 (80%)	

Continuous data are summarized as mean ± standard deviation or median (25^th^ percentile; 75^th^ percentile); SVCS, simple vascular calcification score; HD, Hemodialysis, Hb, hemoglobin; Ca, calcium; P, phosphorus; iPTH, intact parathyroid hormone

^(a)^Pearson Chi-Square test

^(b)^Fisher’s exact test

^(c)^Mann-Whitney test

other p-values for differences between SVCS<3 and SVCS>3 were obtained by were obtained by Student’s t-test.

**Table 2:** Univariable analysis: Association between bone parameters and SVCS.

Variables	All patients (n = 50)	SVCS <3 (n = 30; 60%)	SVCS ≥ 3 (n = 20; 40%)	p value
Bone Volume/Tissue Volume (%)	24.27 ± 8.64	24.92 ± 5.78	23.31 ± 11.83	0.100
Mineralized Bone Volume/Tissue Volume (%)	21.95 ± 7.12	23.56 ± 5.49	19.55 ± 8.63	0.029
Osteoid Volume/Bone Volume (%)	11.09 ± 8.17	12.07 ± 7.82	9.63 ± 8.66	0.437
Osteoid Thickness (μm)	11.42 ± 3.97	11.42 ± 3.11	11.43 ± 5.10	0.989
Mineralization lag time (days)	49.65 ± 42.86	54.36 ± 43.24	42.60 ± 42.40	0.339
Bone formation rate/Bone Surface (mm^3^/cm^2^/year)	2.40 (1.65;4.81)	2.59 (1.72;4.82)	2.17(1.49; 4.77)	0.591^(a)^
Activation frequency (/year)	0.47 (0.27;0.95)	0.27 (.48;.94)	0.47 (0.26; 0.99)	0.968^(a)^
Low bone turnover n (%)	27 (54%)	15 (50%)	12 (60%)	0.481^(b)^
High bone turnover n (%)	19 (38%)	12 (40%)	7 (35%)	0.721^(b)^

Continuous data are summarized as mean ± standard deviation or median (25^th^ percentile; 75^th^ percentile)

^(a)^Mann-Whitney test

^(b)^Pearson Chi-Square test

other p-values for differences between SVCS<3 and SVCS>3 were obtained by Student’s t-test.

### Univariable analysis

Demographic, biochemical and vascular calcification univariable analysis results are shown in Table 1. Among the variables with a p-value < 0.25, gender (p = 0.021), age (p = 0.030) and diabetes (p = 0.007) were the factors associated with SVCS. In the group of patients with SVCS≥3 (Table 1), 12 in 15 patients (80%) had an Agatston score >400 (p<0.001). Diabetes had a very low prevalence in the study sample (n = 5; 10% with 0% in the group of patients with SVCS<3), and was not included in the multivariable analysis. The variable gender was also not included due to the low number of events in the female gender.

Results of bone parameters are shown in Table 2. Lower mineralized bone volume (p = 0.029) was associated with a SVCS ≥3. We found no association between cancellous bone volume, osteoid volume, osteoid thickness, mineralization lag time, bone formation rate, activation frequency and high or low bone turnover with SVCS results.

### Multivariable analysis

**Fig 2:**
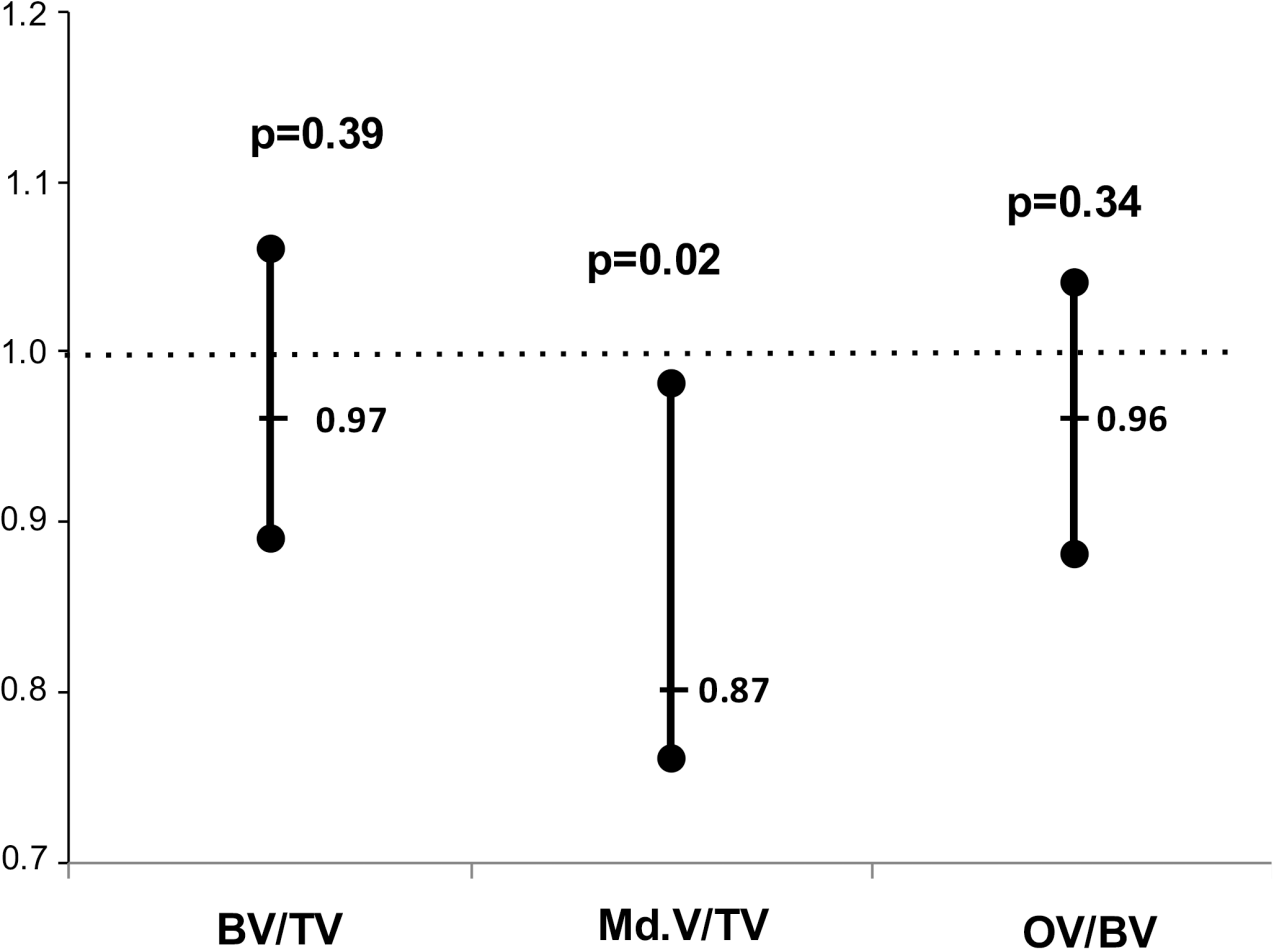
Bone volume and SVCS≥3. Estimated odds ratios of the association of Bone Volume, Mineralized Bone Volume and Osteoid Volume with SVCS≥3 adjusted for age, hemodialysis duration and gender with corresponding confidence intervals and p-values.

**Fig 3:**
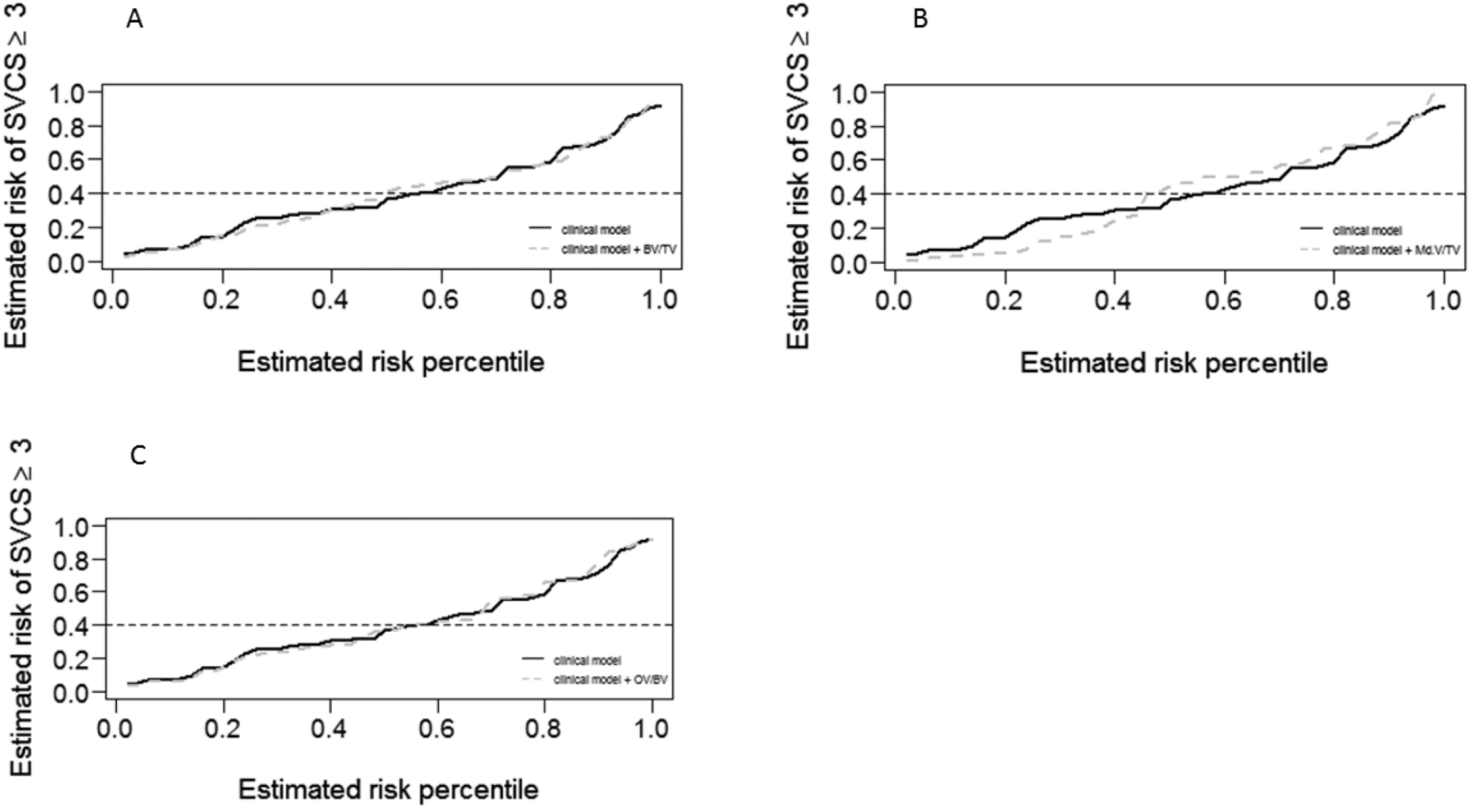
Predictiveness curves. Predictiveness curves corresponding to the clinical model and to the extended model with: **A**- BV/TV; **B**- Md.BV/TV; **C**- OV/BV. The dashed grey line below and above the continuous black line (for lower and higher estimated risks, respectively) shows a best performance only for the model 3, where Md.BV/TV was added to the clinical model.

In the multivariable analysis (Table 3 and Fig 2), a first model (Model 1) was obtained where older age (p = 0.015) and longer hemodialysis duration (p = 0.017) were associated with a SVCS≥3. From all models presented in Table 3, with different bone volume variables, only mineralized bone volume, (p = 0.015) was independently associated with a SVCS≥3. Bone volume consists of mineralized bone volume and osteoid volume. For each increase of 1% in mineralized bone volume there was a 13.7% decrease in the odds of having SVCS≥3 (p = 0.015). The addition of mineralized volume to Model 1 resulted in an increased performance of the model. The performance measures of the multivariable regression logistic models are presented in supplemental Table A (S1A Table). When comparing model 1 and models 2 to 4, using the predictiveness curves, model 3 (model 1 + mineralized bone volume) presented a better performance (Fig 3)

**Table 3:** Multivariable logistic regression models. Binary dependent variable: (SVCS<3, SVCS≥3).

**Model 1**			
	OR	95% CI	p value
age	1.066	1.013–1.126	0.015
HD duration*	1.131	1.023–1.250	0.017
**Model 2 = Model 1 + Bone Volume**			
age	1.065	1.011–1.121	0.018
HD duration*	1.142	1.028–1.268	0.013
Bone Volume	0.965	0.888–1.049	0.404
**Model 3 = Model 1 + Mineralized Bone Volume**			
age	1.068	1.006–1.133	0.030
HD duration*	1.214	1.060–1.390	0.005
**Mineralized Bone volume**	0.863	0.766–0.971	**0.015**
**Model 4 = Model 1 + Osteoid Volume**			
age	1.064	1.010–1.121	0.020
HD duration*	1.142	1.031–1.265	0.011
Osteoid Volume	0.952	0.878–1.033	0.236

OR, odds ratio estimate; HD, hemodialysis; Bone Volume consists of Mineralized Bone Volume and Osteoid Volume. The different models apply to the same population (n = 50).

*Increased odds of SVCS≥3 for each six-month increase in HD duration.

### Agreement between SVCS and Agatston score

The study of agreement between SVCS and Agatston score resulted in a Spearman’ s correlation coefficient estimate of 0.71 (95%CI: 0.51, 0.86).

**Fig 4:**
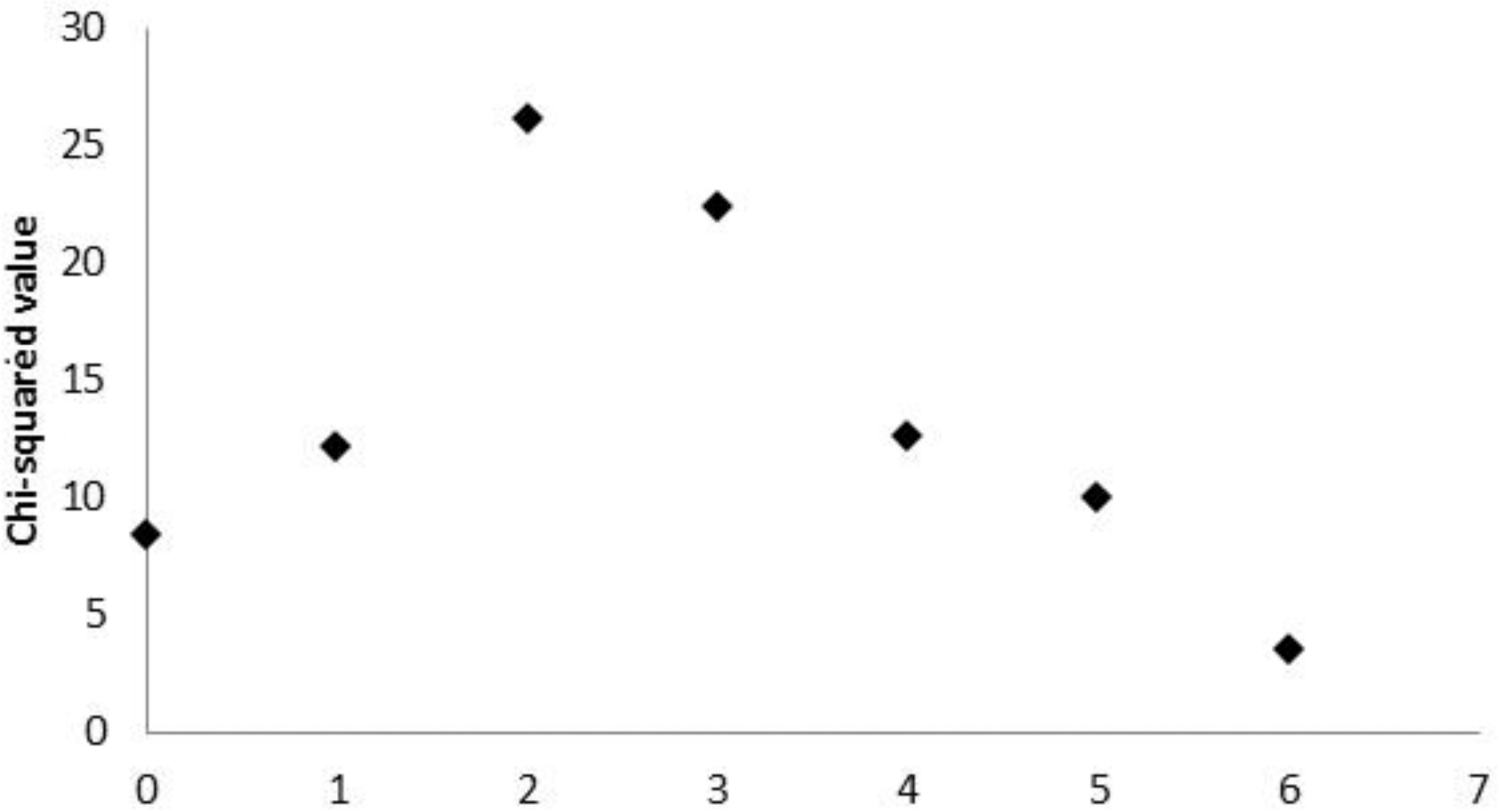
Cut-point for SVCS. Chi-square values measuring the association between binary Agatston score and dichotomized SVCS (at potential cut-points). The maximum chi-squared value occurs at SVCS = 2, enabling the dichotomization of this variable as SVCS<3 and ≥3.

A complementary study of agreement was additionally performed after dichotomizing both variables. Regarding Agatston score, the cut-point 400 was considered (40), and results of the minimum p-value approach (Fig 3) identified a SVCS greater than 2 as the best cut-point, enabling the discretization of this variable as SVCS<3 and SVCS≥3 (Fig 4). This result is in accordance with the cut-point previously obtained for higher cardiovascular mortality risk (3,18).

In the group of patients with SVCS≥3 (Table 1), 12 of 15 patients (80%) had an Agatston score >400 (p<0.001), and a Cohen’s kappa of 0.79 (95%CI: 0.59, 0.99) was found, indicating a substantial agreement between the two binary SVCS and Agatston scores (38,39). Additionally, a sensitivity of 0.92 (95%CI: 0.64, 1.00), a specificity of 0.90 (95%CI: 0.73, 0.98), a positive predictive value of 0.80 (95%CI: 0.55, 0.99), and a negative predictive value of 0.96 (95%CI: 0.79, 0.99), were obtained. The AUC achieved by this binary SVCS score regarding the discrimination of the patients with Agatston score>400 was 0.92 (95%CI: 0.80, 1.00), indicating an appropriate discrimination ability.

## Discussion

In 2006, KDIGO included vascular calcifications in the diagnosis of chronic kidney disease mineral and bone disorder (CKD-MBD) [11]. The evaluation of the relationship between bone histomorphometric abnormalities and vascular calcifications was one of the suggested KDIGO questions for clinical research.

We demonstrate in this study an association between higher mineralized bone volume with a lower vascular calcification score evaluated by plain X-ray. [3].

Bone is the main reservoir of calcium and phosphate in the organism and acts as a mineral pool for calcium homeostasis [42]. Bone volume is composed of mineralized bone and non-mineralized osteoid bone. Hydroxyapatite, a crystalline complex of calcium and phosphate, constitutes 70% of mineralized bone [42]. The maintenance of the body’s steady state of calcium and phosphorus is the result of the concerted action of intestinal, renal and skeletal regulatory mechanisms under tight hormonal control [43]. In dialysis patients, according to the Braun model [44], bone has a decreased capacity to buffer high blood levels of calcium and phosphate and, in association with oligo / anuria with reduced calciuria and phosphaturia, the only escape is the development of vascular calcifications.

In dialysis patients several studies have shown an association between bone disease and vascular calcifications. Low bone turnover [12,14], changes in bone remodelling [13], low bone volume [15] have been associated with vascular calcifications diagnosed by ultrasonography or multislice computed tomography. Bone fractures have been associated with vascular calcifications evaluated by plain X-ray [17]. Adynamic bone disease has been associated with progression of coronary calcifications [13]. Lower bone mineral density has been associated with arterial stiffness [45,46], and with progression of coronary calcifications [16].

The high prevalence of both low bone volume and low bone turnover has been demonstrated in a study evaluating 630 bone biopsies from dialysis patients [16]. Low bone formation is the mechanism that links low bone volume to low bone turnover [16]. Our finding of the association of higher mineralized bone volume with a lower vascular calcifications score is also in agreement with previous findings of the association of low bone turnover with higher vascular calcifications scores [12–14]. We expand this observation by identifying mineralized bone as the major factor of association.

We have previously demonstrated an association between low bone volume with coronary calcifications [15]. In the present study we have verified an association between the simple vascular calcification score evaluated in plain X-ray [3] with mineralized bone volume. Our results highlight the association of mineralized bone volume with vascular calcifications, calling attention for the role of mineralized bone as reservoir of calcium and phosphate and its possible contribution for the burden of vascular calcifications in dialysis patients.

Computed tomography is the most sensitive method to evaluate the prevalence and progression of vascular calcifications but, due to the higher cost and the higher radiation dose it is not suitable to be used in the daily clinical routine.

The SVCS is inexpensive, very easy to interpret by the Nephrologist, widely available, and uses a very low radiation dose. In CKD patients, this score has been associated with all-cause and cardiovascular mortality [3,18–20], with arterial stiffness [18,46], with low or high ankle brachial-index [47] and with low bone mineral density [46]. In a large cohort of CKD patients not on dialysis, this SVCS, but not the Kauppila score, was associated with all-cause and cardiovascular mortality [20]. We have also verified in the present study that there is a substantial agreement between an Agatston score > 400 and a SVCS ≥ 3.

The main limitation of this study is its observational nature that allows only to show associations and no cause and effect relationship. The low number of diabetic patients in this study did not allow to address diabetes as a risk factor. The strength of this study lies in the availability of bone biopsies for direct tissue analysis.

In summary, our analysis shows an association of higher mineralized bone volume evaluated in bone biopsies, with a lower vascular calcification score assessed by plain X-ray. The decreased risk of the association of higher mineralized bone volume with a lower vascular calcification score suggests that there might be a role for bone in the reduction of the high cardiovascular risk in hemodialysis patients. This study reinforces the utility of this simple plain x-ray method to evaluate vascular calcifications for dialysis patients and supports the hypothesis of the existence of a link between bone mineralization and vascular calcification. If this hypothesis is correct, it is possible that a therapeutic intervention in bone disease could have an impact on patient cardiovascular outcomes beyond the beneficial effect on bone.

## Supporting information

S1 Table Performance of the multivariable regression logistic models. (DOCX)
