# Supplementary material for: Higher mineralized bone volume is associated with a lower plain X-Ray vascular calcification score in hemodialysis patients
Source: PLoS One. 2017 Jul 7;12(7):e0179868. doi: 10.1371/journal.pone.0179868 (PMC5501435; doi:10.1371/journal.pone.0179868)
Supplement: S1 Table — (DOCX) [file pone.0179868.s001.docx]

**Statistical analysis – Supplemental information**

The multivariable analysis was performed using logistic regression models. To obtain a clinical model (model 1), all the variables with a p-value <0.25 obtained in the univariable analysis and with clinical relevance, were considered. To compare bone parameters regarding their association with vascular calcifications measured by SVCS, four additional models were adjusted. In order to quantify the improvement resulting from adding BV/TV (Model 2), Md.V/TV (Model 3) and OV/BV (Model 4) to a clinical model (Model 1), continuous net reclassification improvement (NRI) and integrated discrimination improvement (IDI) measures were calculated. Predictiveness curves were also calculated [1].

These models were evaluated regarding: overall performance by using Nagelkerke R^2^ and Brier score, calibration assessed by the Hosmer-Lemeshow test, and discrimination by calculating the area under the receiver-operating characteristic curve (AUC).

Box-Tidwell transformation to test the assumption of linearity in the logit for continuous variables was used. Predictiveness curves were also calculated.

[2,3]. The NRI quantifies the correctness of upward and downward movement of predicted probabilities as a result of adding a new marker to an existing baseline model. The IDI quantifies the magnitudes of changes in these probabilities. Predictiveness curves are graphical representations of the distribution of risk defined by the following conditional probability: risk(y)=P[D=1|Y=y], where D denotes the binary outcome (in our case, SVCS ≥3) and Y=y is in our case the estimated probabilities of having a SVCS ≥3. The interpretation of this curve is straightforward: a marker that is useless assigns equal risk to all individuals, and hence, the corresponding predictiveness curve is a horizontal line at the prevalence of the disease; however, a marker that is highly informative about risk yields a predictive curve that is close to a step function.

.Huang Y, Sullivan Pepe M, Feng Z. Evaluating the predictiveness Evaluating the predictiveness of a continuous marker. *Biometrics* 63(4):1181-8, 2007.

2.Pencina MJ, D'Agostino RB Sr, Steyerberg EW. Extensions of net reclassification improvement calculations to measure usefulness of new biomarkers. *Stat Med* 30(1): 11-21, 2011.

3.Pencina MJ, D'Agostino RB Sr, D'Agostino RB Jr, Vasan RS. Evaluating the added predictive ability of a new marker: from area under the ROC curve to reclassification and beyond*. Stat Med* 27(2):157-72; discussion 207-12, 2008.

**Supplemental Table 1. Performance of the Multivariable Regression Logistic Models**

| Performance measures | MODEL 1 | MODEL 2 | MODEL 3 | MODEL 4 |
| --- | --- | --- | --- | --- |
| OVERALL PERFORMANCE |  |  |  |  |
| R^2^ (Nagelkerke) (%) | 31.0% | 32.6% | 45.6% | 34.0% |
| Brier Score | 0.190 | 0.184 | 0.155 | 0.182 |
| DISCRIMINATION  AUC  (CI) | 0.770  (0.642, 0.898) | 0.777  (0.647, 0.907) | 0.832  (0.719, 0.944) | 0.787  (0.662, 0.912) |
| CALIBRATION |  |  |  |  |
| Hosmer Lemeshow test p-value | 0.784 | 0.641 | 0.672 | 0.615 |
| ADDED VALUE ^(a)^ |  |  |  |  |
| NRI, %(CI) |  | 60.0 (3.4, 116.6) | 70.0 (13.4, 126.6) | 26.7 (-29.9, 83.2) |
| NRI events, %(CI) |  | 40.0 (-3.8, 83.8) | 30.0 (-13.8, 73.8) | 0.0 (-43.8, 43.8) |
| NRI non-events, %(CI) |  | 20.0 (-15.8, 55.8) | 40.0 (4.2, 75.8) | 26.7 (-9.12, 62.5) |
|  |  |  |  |  |
| IDI |  | 0.018 (-0.017, 0.054) | 0.130 (0.029, 0.231) | 0.029 (-0.017, 0.075) |
| IDI events |  | 0.011 (-0.021, 0.043) | 0.078 (-0.010, 0.166) | 0.017 (-0.019, 0.054) |
| IDI non-events |  | 0.007 (-0.008, 0.023) | 0.052 (0.001, 0.103) | 0.012 (-0.017, 0.040) |
